# *Borrelia burgdorferi* Enolase Is a Surface-Exposed Plasminogen Binding Protein

**DOI:** 10.1371/journal.pone.0027502

**Published:** 2011-11-08

**Authors:** Angela M. Floden, John A. Watt, Catherine A. Brissette

**Affiliations:** 1 Departments of Microbiology and Immunology, University of North Dakota School of Medicine and Health Sciences, Grand Forks, North Dakota, United States of America; 2 Departments of Anatomy and Cell Biology, University of North Dakota School of Medicine and Health Sciences, Grand Forks, North Dakota, United States of America; University of Louisville, United States of America

## Abstract

*Borrelia burgdorferi* is the causative agent of Lyme disease, the most commonly reported arthropod-borne disease in the United States. *B. burgdorferi* is a highly invasive bacterium, yet lacks extracellular protease activity. In order to aid in its dissemination, *B. burgdorferi* binds plasminogen, a component of the hosts' fibrinolytic system. Plasminogen bound to the surface of *B. burgdorferi* can then be activated to the protease plasmin, facilitating the bacterium's penetration of endothelial cell layers and degradation of extracellular matrix components. Enolases are highly conserved proteins with no sorting sequences or lipoprotein anchor sites, yet many bacteria have enolases bound to their outer surfaces. *B. burgdorferi* enolase is both a cytoplasmic and membrane associated protein. Enolases from other pathogenic bacteria are known to bind plasminogen. We confirmed the surface localization of *B. burgdorferi* enolase by *in situ* protease degradation assay and immunoelectron microscopy. We then demonstrated that *B. burgdorferi* enolase binds plasminogen in a dose-dependent manner. Lysine residues were critical for binding of plasminogen to enolase, as the lysine analog εaminocaproic acid significantly inhibited binding. Ionic interactions did not play a significant role in plasminogen binding by enolase, as excess NaCl had no effects on the interaction. Plasminogen bound to recombinant enolase could be converted to active plasmin. We conclude that *B. burgdorferi* enolase is a moonlighting cytoplasmic protein which also associates with the bacterial outer surface and facilitates binding to host plasminogen.

## Introduction

*Borrelia burgdorferi,* the Lyme disease spirochete, causes the most common arthropod-borne disease in the United States and many other temperate regions of the world. Lyme disease is a significant cause of morbidity and it continues to be a serious public health concern [1]. Spirochetes such as *B. burgdorferi* are unique pathogens in that they lack classical virulence factors such as toxins. Instead, part of their remarkable ability to cause disease and suffering lies in their ability to widely disseminate throughout host tissues. *B. burgdorferi* lacks surface proteases that could degrade the hosts' extracellular matrix and accelerate the spirochete's penetration of host tissues. However, *B. burgdorferi* can usurp a host protease, plasminogen.

Plasminogen is a serine protease present in serum as an inactive proenzyme [2]. Plasminogen is converted by tissue-type plasminogen activator (tPA) or urokinase plasminogen activator (uPA) to active plasmin. Plasmin has a critical role in host fibrinolysis and extracellular matrix remodeling and therefore its activity is tightly controlled. Binding of plasminogen by mammalian plasminogen receptors is mediated by lysine-binding Kringle domains [3]. Binding of plasminogen to a mammalian receptor, fibrin clot, or a bacterial cell facilitates its activation to plasmin and makes the molecule less susceptible to inactivation by α2 antiplasmin [4], [5].

Many bacterial species are able to bind and use host plasminogen. *B. burgdorferi* is known to bind host plasminogen, which can then be converted to active plasmin. *B. burgdorferi* with bound plasmin is able to degrade fibronectin, penetrate the endothelium, and activate matrix metalloprotease-9 (MMP-9) and MMP-1 [6], [7], [8]. Plasminogen is required for efficient dissemination in ticks, and plasminogen deficient mice have decreased spirochetemia [9]. Several plasminogen-binding proteins have been identified and characterized in *B. burgdorferi,* including ErpA/C/P, OspA, OspC, and a 70 kDa plasminogen binding protein [10], [11], [12], [13].

Enolases are cytosolic metalloenzymes that catalyze the conversion of 2-phospho-D-glycerate to phosphoenolpyruvate [14]. Despite the lack of classical protein sorting machinery or cell membrane anchoring moieties, enolases are expressed on the surface of a variety of eukaryotic cells (including neuronal, cancer, epithelial, endothelial and hematopoietic cells), where they can function as plasminogen receptors [14]. Remarkably, enolases are also found on the surface of bacterial cells where they can similarly function as plasminogen receptors [4], [14], [15], [16].

While several plasminogen-binding proteins have been proposed and partially characterized for *B. burgdorferi*, we reasoned that the *B. burgdorferi* enolase might also be a plasminogen-binding protein. We now demonstrate that the *B. burgdorferi* enolase is exposed on the bacterial outer surface, and can bind host plasminogen.

## Materials and Methods

### Bacteria

Virulent *Borrelia burgdorferi* strain B31-MI-16 [17], [18], [19] was grown at 34°C to cell densities of approximately 1×10^7^ ml in modified Barbour-Stoenner-Kelly II (BSK-II) medium [20]. Total DNA (chromosomal and plasmids) was isolated using the DNeasy blood and tissue kit (Qiagen, Valencia, CA) according to the manufacturer's instructions.

### Recombinant proteins

Polyhistidine-tagged, full-length ErpC has been described previously [10], [21], [22]. All recombinant proteins contained amino-terminal tags. The enolase gene (ORF BB0337) plus approximately 500 bp upstream and downstream DNA, was produced by PCR of *B. burgdorferi* B31-MI-16 DNA using primers enoF flk 5′ tgcttgtgccatgaggaata and enoR flk 5′ ataaggcacggcatttcaag and subsequent cloning into pCR2.1 (Invitrogen, Carlsbad, CA). Full-length enolase for recombinant protein production was produced by PCR using primers enoF prot 5′caccatgggttttcacatttatgaaatca and enoR prot 5′ttatttttgtttaatagaataaaagacgctc and insertion into pET200 (Invitrogen). The resultant plasmid's insert was entirely sequenced on both strands to ensure that no undesired mutations had occurred during PCR or cloning procedures. Recombinant proteins were expressed in *Escherichia coli* Rosetta (DE3)pLysS (Novagen, Madison, WI), upon induction with isopropyl-β-D-thiogalactopyranoside (IPTG). Induced *E. coli* cells were harvested and then lysed by two passages through a French pressure cell at 1,000 p.s.i in a mixture of 30 mM imidazole, 0.5 M NaCl, and 20 mM NaPO_4_ (pH 7.4), and debris was cleared by centrifugation. Recombinant proteins were purified from cleared lysates using MagneHis nickel-conjugated magnetic beads (Promega, Madison, WI). All recombinant proteins were dialyzed overnight against phosphate-buffered saline (PBS) using 3,500 kDa molecular-weight-cutoff Slide-A-Lyzer cassettes (Pierce, Rockford, IL) at 4°C. Protein purity was assessed by sodium dodecyl sulfate-polyacrylamide gel electrophoresis (SDS-PAGE) followed by staining with Coomassie brilliant blue. Protein concentrations were determined by bicinchoninic acid protein assays (BCA; Pierce).

### SDS-PAGE and Western blotting

Fresh BSKII medium and spent BSKII medium from late log phase cultures of *B. burgdorferi* were centrifuged briefly (12,000 x g, 10 minutes) and the supernatants, recombinant enolase (1.25 µg/ml) or whole-cell lysate from log-phase *B. burgdorferi* (2×10^7^ bacteria/ml) were separated by SDS-PAGE and proteins were either transferred to nitrocellulose membranes or stained with Coomassie brilliant blue. Membranes were blocked overnight at 4°C with 5% (w/v) non-fat dried milk in Tris-buffered saline-Tween 20 (TBS-T; 20 mM Tris (pH7.5), 150 mM NaCl, 0.05% (v/v) Tween 20). Membranes were next washed with TBS-T, and incubated for 2 h at room temperature with rabbit antiserum raised against human α-enolase (Pierce) diluted 1∶500 in TBS-T. After extensive washing with TBS-T, membranes were incubated for 1 h at room temperature with horseradish-peroxidase-conjugated donkey anti-rabbit IgG antibody (GE Healthcare, Piscataway, NJ), diluted 1: 20,000 in TBS-T. After a final series of washes with TBS-T, bound antibodies were detected using SuperSignal West Pico enhanced chemiluminescence substrate (Pierce).

### *In situ* protease analysis

*B. burgdorferi* were grown to mid-exponential phase in BSK-II, pelleted by centrifugation, washed once with PBS, and resuspended in PBS to a final concentration of approximately 2×10^9^ bacteria/ml. Examination of bacterial suspensions by darkfield microscopy did not indicate detectable lysis of the bacteria. Bacteria were then incubated at room temperature in PBS containing 40 µg of proteinase K (Fisher Scientific, Pittsburgh, PA) for 30, 60 or 120 minutes, whereupon digestion was terminated by addition of paramethylsulfonyl fluoride (PMSF; Sigma) to a final concentration of 1.6 mg/ml, followed by sample boiling. Control aliquots of bacteria were incubated in buffer for 2 h at room temperature without added protease, followed by the addition of inhibitor and boiling as with the protease-treated bacteria. Equal volumes of each bacterial lysate were subjected to SDS-PAGE and transferred to nitrocellulose membranes, and the susceptibility of enolase to protease digestion was assessed by immunoblot analysis with rabbit polyclonal anti-human α-enolase (Pierce) as described above. As experimental controls, lysates were also immunoblotted with monoclonal antibodies directed against OspC (located on the bacterial outer surface and thus susceptible to proteolysis [22], [23]) and FlaB (located in the periplasmic space and thus protected against protease digestion in intact bacteria [24]). Densitometric analysis was performed using ImageJ software (http://imageJ.nih.gov/ij).

### Immunoelectron microscopy

For immunogold localization of enolase and OspC, cultures of *B. burgdorferi* were grown to mid-exponential phase then harvested by centrifugation and washed three times in sterile PBS, pH 7.4. TEM grids were washed repeatedly in PBS both before processing and between all incubation steps. All solutions were microfiltered using 0.4 µm syringe filters prior to use. Fifty microliter droplets containing non-diluted *B. burgdorferi.* were placed on the surface of parafilm covered plastic petri dishes. Gold 400 mesh formvar coated carbon-stabilized TEM grids (Ted Pella, Redding, CA) were then placed on each droplet for 90 mins at room temperature followed by a 20 minute fixation with buffered 4% paraformaldehyde. Sections were then incubated sequentially in the appropriate blocking buffer (4% normal serum/PBS, 1 hr. at room temperature), polyclonal rabbit anti-human α-enolase (1∶50 in blocking buffer, Pierce) or monoclonal mouse anti-OspC ([25] kind gift of Brian Stevenson), for 2 hours followed by biotinylated goat anti-rabbit or horse anti mouse IgG (1∶500 in blocking buffer, 2 hr.; Vector Laboratories, Burlingame, CA), respectively. This was followed by a 30 min incubation on droplets of streptavidin-conjugated 10 nm gold particles (Ted Pella). Grids were stabilized by post-fixation on droplets of 4% paraformaldehyde/2% glutaraldehyde for 10 minutes and washed repeatedly in PBS. To further assess specificity of the monoclonal anti-enolase antibody, additional spirochete grids were processed as described above with omission of the primary antibody. Grids were then counterstained by placing on droplets of a 5% molybdate solution for 60 seconds, washed repeatedly in PBS and viewed using a Hitachi 7500 transmission electron microscope.

### ELISA

For the enzyme-linked immunosorbent assay (ELISA), Maxisorp 96- well plates (Nalge-Nunc, Rochester, NY) were coated overnight with 10 µg/ml human plasminogen (Sigma-Aldrich, St. Louis, MO), 10 µg/ml recombinant enolase, 10 µγ/ml recombinant ErpC (positive control for plasminogen binding), or 10 µg/ml bovine serum albumin (BSA [negative control]) in PBS at 4°C. Plates were brought to room temperature and washed once with PBS supplemented with 0.05% (vol/vol) Tween 20 (PBS-T). Wells were blocked for 2 h at room temperature with PBS with 1% (mass/vol) gelatin and then washed three times with PBS-T. Afterwards, 100 µl/well of either recombinant enolase, ErpC or plasminogen (Sigma-Aldrich) [see text for concentrations]) was added and incubated for 2 h at 37°C. Wells were washed three times with PBS-T and then incubated for 1 h at room temperature with rabbit antiserum raised against human α-enolase (which cross-reacts with the highly similar *Borrelia burgdorferi* protein; Pierce), diluted 1∶500 in PBS, or goat anti-human plasminogen (Novus Biologicals, Littleton, CO). Plates were washed three times with PBS-T, and then wells were incubated for 1 h at room temperature with horseradish peroxidase-conjugated anti-goat immunoglobulin G (IgG; Jackson Immunochemicals, West Grove, PA), horseradish peroxidase-conjugated anti-rabbit IgG (GE Healthcare), or horseradish peroxidase-conjugated protein G (Invitrogen), diluted 1∶5,000. Wells were again washed three times with PBS-T, 100 µl/well. Tetramethylbenzidine substrate (Thermo Scientific, Rockford, IL) was added, and then reactions were stopped by addition of 100 µl/well of 2 N H_2_SO_4_. Absorbance was read at 450 nm using a BioTek plate reader utilizing KC4 software (BioTek, Winooski, VT). To determine the role of lysines in plasminogen-enolase interaction, the lysine analog ε-aminocaproic acid (final concentration, 3–30 mM [Sigma-Aldrich]) was added with plasminogen to enolase-coated wells. Protein binding affinities (*Kd*) were calculated as that concentration of ligand required for half-maximal binding activity. For experiments examining the role of ionic interactions in enolase binding to plasminogen, increasing concentrations of NaCl were added to the PBS-based binding buffer with enolase. For experiments analyzing the ability of whole *B. burgdorferi* to bind recombinant enolase, plates were coated overnight with intact *B. burgdorferi* (2×10^7^/ml; washed 3X in PBS); after washing and blocking with SeaBlock (Pierce) for 2 hours, recombinant *B. burgdorferi* enolase (0–50 µg/ml) was added for 2 hours at 37°C, plates washed, and enolase detected as described above.

### Plasminogen activation assay

Maxisorp 96-well plates (Nalge-Nunc) were coated overnight with 10 µg/ml recombinant enolase or 10 µg/ml BSA in PBS at 4°C. Plates were brought to room temperature and washed once with PBS-T. Wells were blocked for 2 h at room temperature with PBS with 2% (mass/vol) BSA and then washed three times with PBS-T. Afterwards 100 µl/well of 10 µg/ml human plasminogen was added and incubated for 2 h at 37°C. Wells were washed three times with PBS-T, and then 4 ng/well of human uPA (Chemicon, Temecula, CA) was added. Next, the plasmin-specific substrate D-valyl-leucyl-lysine-*p*-nitroanilide dihydrochloride (Sigma-Aldrich) was added at a final concentration of 0.3 mM in 64 mM Tris, 350 mM NaCl, 0.15% Triton X-100 (pH 7.5). Plates were incubated overnight at 37°C, and absorbance was read at 405 nm.

## Results

### *B. burgdorferi* enolase is surface exposed

**Figure 1:**
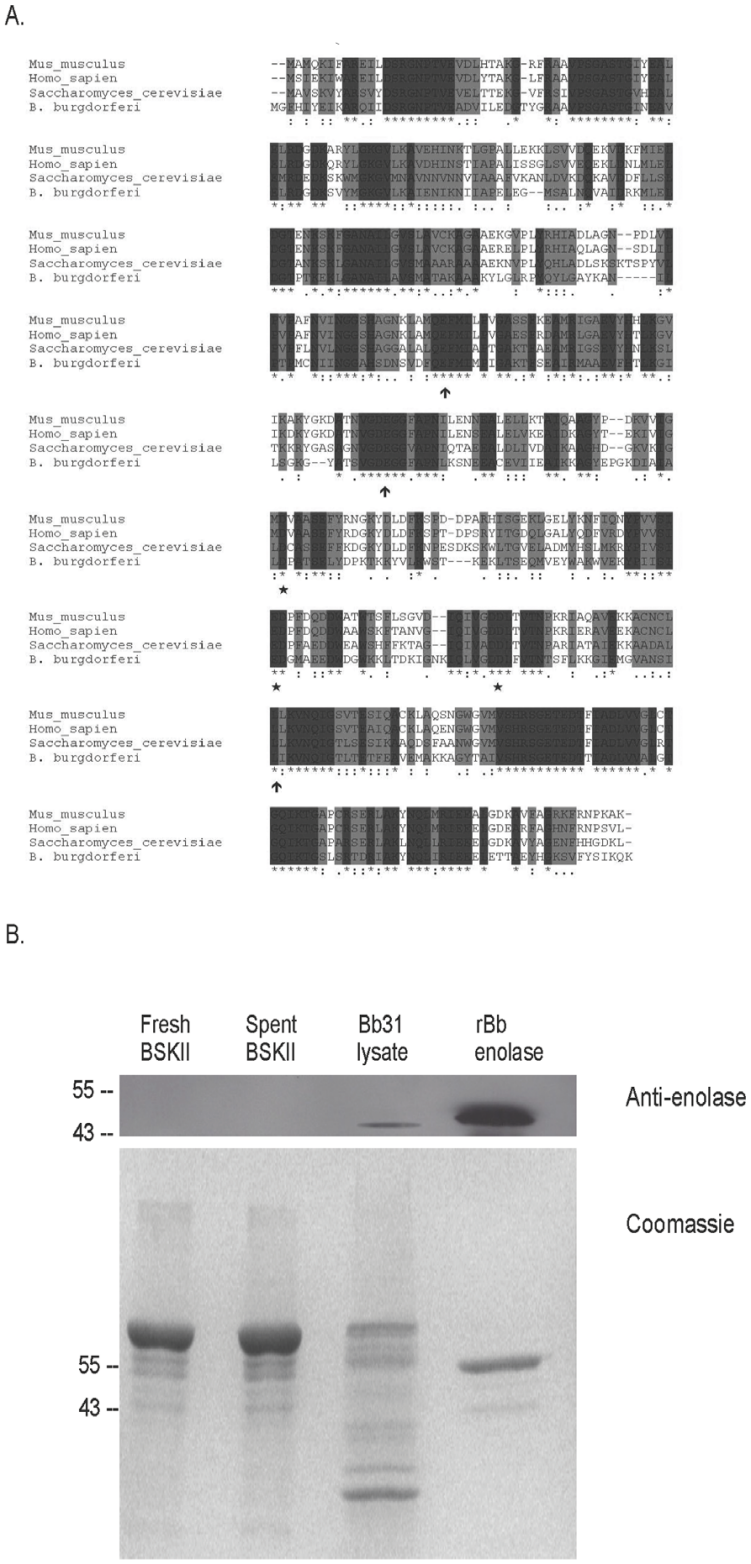
Conservation of enolase. (A) Alignment of enolases from mouse, human, yeast and *B. burgdorferi*. Dark shaded residues indicate identity; light gray residues indicate similarity. Arrows indicate the 3 conserved residues that make up the enzymatic active site; stars indicate the three conserved residues required for binding the cofactor manganese. (B) A rabbit polyclonal antiserum directed against human enolase recognizes enolase from *B. burgdorferi*. Top panel: Western blot; Bottom panel: Coomassie-stained SDS-PAGE gel. Molecular weight markers are indicated on the left. Lane 1: fresh BSKII medium; Lane 2: spent BSKII medium from late-log phase cultures of *B. burgdorferi*; Lane 3: *B. burgdorferi* cell lysate; Lane 4: recombinant enolase from *B. burgdorferi*.

Enolases are highly conserved among organisms as diverse as bacteria and humans [14]. Alignment of enolases from diverse species suggested that an anti-human antibody might recognize *B. burgdorferi* enolase (Figure 1A). Notably, the amino acid residues required for binding the cofactor, manganese (165E, 206E, 339L), as well as the residues of the enzymatic active site (243D, 289E, 317D), were also conserved (Figure 1A and [27]). Recombinant *B. burgdorferi* enolase or whole cell lysate were separated by SDS-PAGE and transferred to nitrocellulose. As anticipated, an anti-human α-enolase polyclonal antibody recognized purified recombinant *B. burgdorferi* enolase as well as the native enolase in whole cell lysates (Figure 1B). Since the *B. burgdorferi* medium is rich in bovine serum albumin and rabbit serum, we confirmed that the medium itself was not recognized by the anti-human α-enolase antibody (Figure 1B, fresh BSK-II lane).

**Figure 2:**
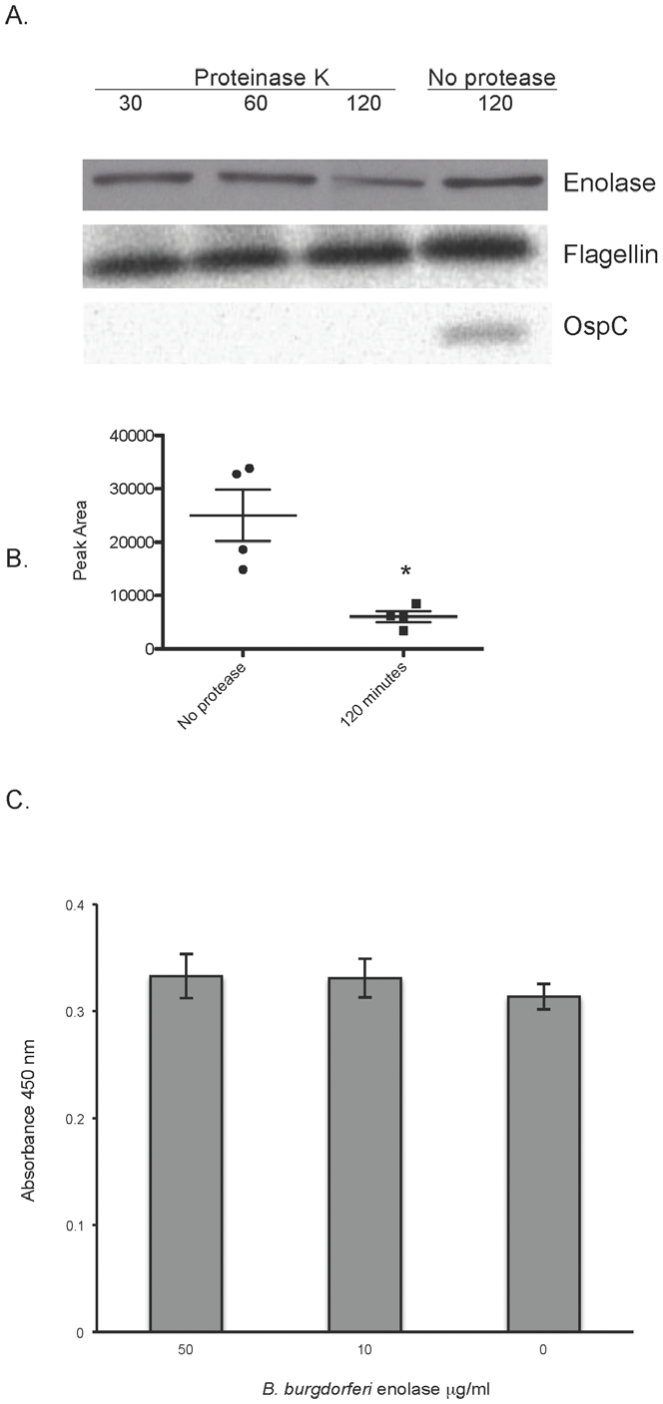
*B. burgdorferi* enolase is surface exposed. (A) Demonstration of outer surface exposure of enolase by *in situ* protease degradation. Whole *B. burgdorferi* were incubated with proteinase K for 30, 60 or 120 minutes or 120 minutes in buffer with no protease. Proteases were then inactivated, bacteria were lysed, proteins were separated by SDS-PAGE, and the integrity of enolase, OspC (outer surface protein), and FlaB (periplasmic protein) were analyzed by immunoblot. Two separate protease degradation experiments were performed, with blots repeated 4 times. A representative blot is shown. (B) Densitometric analysis of *in situ* protease degradation immunoblots. Images from blots were scanned and peak areas for 120-minute proteinase K treatment and no protease control were assessed using ImageJ software (http://imageJ.nih.gov/ij). Data represent the means and standard errors from 4 separate blots. *, *P* = 0.03, Student's *t* test assuming unequal variances. (C) Binding of exogenous *B. burgdorferi* enolase to the surface of whole bacteria. Binding of *B. burgdorferi* enolase (0–50 µg/ml) to immobilized *B. burgdorferi* (2×10^7^/ml) was analyzed by ELISA, with bound enolase detected by rabbit polyclonal antiserum directed against human enolase. Data represent the means and standard errors from three separate experiments with six replicates per enolase concentration.

Despite the lack of canonical protein export sequences, enolases of many species, both bacteria and eukaryote, are localized on the cell surface [14], [16], [27], [28], [29], [30], [31], [32], [33], [34], [35]. The *B. burgdorferi* enolase was previously shown to fractionate with both the soluble and membrane fractions [36]. To address the possibility of surface exposure of enolase, *in situ* protease degradation assays were performed. Intact *B. burgdorferi* were incubated in the presence of proteinase K for 30, 60 or 120 minutes. Protease was then inactivated, bacteria were lysed and proteins separated by SDS-PAGE. After transfer to nitrocellulose membranes, blots were incubated with antisera specific for OspC, an outer surface protein, FlaB, a periplasmic protein, or enolase. As shown in Figure 2A, OspC was totally degraded, indicating surface exposure. FlaB was not affected by the protease, indicating that the bacteria were intact. There was a considerable reduction in the amount of enolase detected by 120 minutes of incubation with proteinase K, compared to the no protease control. Densitometric analysis of band intensities revealed a significant reduction in peak area between the 120-minute proteinase K treated band and the no protease control (Figure 2B). Although enolase is a glycolytic enzyme, and found within the cytoplasm, these data indicate that a proportion of *B. burgdorferi* enolase is also surface exposed.

In Gram-positive bacteria such as *Streptococcus pneumoniae*, it has been suggested that autolysins are involved in liberating enolase from the bacterial cytoplasm, and that streptococci scavenge enolase from lysed neighbor cells [37], [38]. However, we were unable to detect enolase in spent culture medium from late log phase *B. burgdorferi* (Figure 1B, spent BSKII lane). In addition, we incubated intact *B. burgdorferi* (2×10^7^ bacteria/ml) with recombinant *B. burgdorferi* enolase and assayed the amount of surface-associated enolase by ELISA. We did not detect any significant increase in surface-bound enolase compared to bacteria incubated in the absence of exogenous *B. burgdorferi* enolase (Figure 2C).

**Figure 3:**
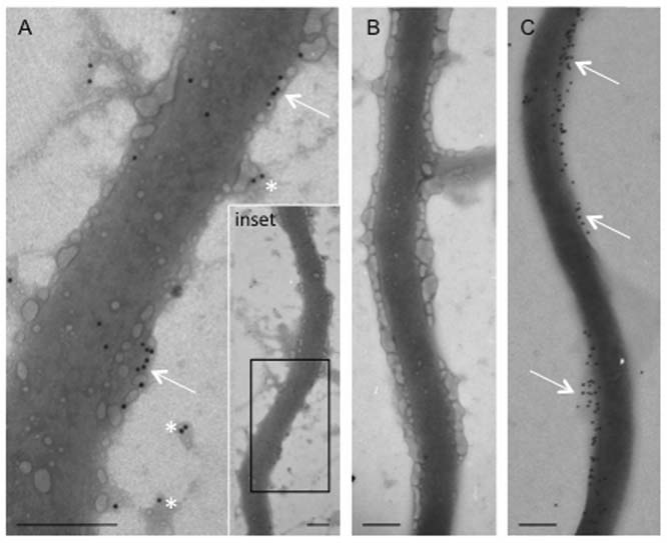
Immunoelectron microscopic analysis of *B. burgdorferi* enolase. A) Anti-enolase was localized intermittently across the outer surface of *B. burgdorferi (arrows)*. Gold particles were also observed on membrane blebs in proximity to the spirochete (asterisks). The boxed area in the inset indicates the region demonstrated in image (A). B) Omission of the primary antibody resulted in a complete loss of immunoreactivity. C) Anti-OspC immunolabeling demonstrated moderately heavy labeling on the outer surface of the spirochete (Arrows). Magnification bar  = 0.2 µm.

We then used immunoelectron microscopy to confirm the presence of enolase on the spirochete outer membrane. Our analysis revealed that gold-labeled anti-human α-enolase was scattered sporadically over the outer surface of the majority of spirochetes (Figure 3A). The density of labeling was quite variable cell-to-cell suggesting some variability in surface exposure of enolase among individual spirochetes. It is noteworthy that labeling was routinely observed on isolated patches of material resembling outer membrane (Figure 3A. asterisks). Omission of the anti-enolase antibody resulted in the complete absence of gold labeling on all control grids examined (Figure 3B). Antibody against an abundant outer surface protein, OspC, was used as a positive control. Anti-OspC labeling was generally denser than enolase, and tended to localize in large patches covering the surface of the spirochete (Figure 3C). The dense labeling of OspC is consistent with the presence of a well-preserved outer membrane and supports our data indicating the localization of enolase to the outer surface of *B. burgdorferi*.

### Enolase binds human plasminogen

**Figure 4:**
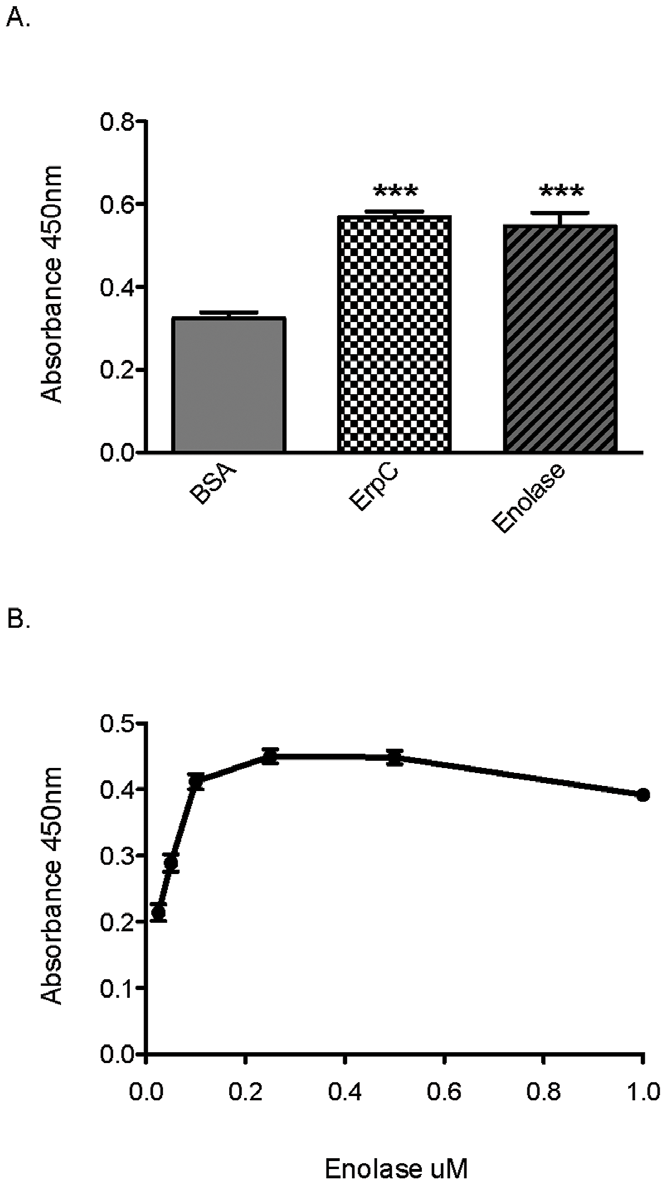
*B. burgdorferi* enolase binds plasminogen. (A) Binding of plasminogen (25 µg/ml) to immobilized proteins (10 µg/ml) was analyzed by ELISA, with bound plasminogen detected using specific antiserum. BSA was used as a negative control for nonspecific binding. ErpC is a known plasminogen binding protein of *B. burgdorferi* [10]. Data represent the means and standard errors from three separate experiments with six replicates per protein. ***, *P*<0.001, Student's *t* test assuming unequal variances. (B) Enolase binds plasminogen in a dose-dependent manner. Binding of plasminogen (10 µg/ml) to immobilized enolase (0–1 µM) was analyzed by ELISA, with bound plasminogen detected using specific antiserum. BSA was used as a negative control for nonspecific binding. Values represent plasminogen binding to enolase minus background absorbance for BSA. Data represent the means and standard errors from a representative experiment (1 of 4) with six replicates per concentration of enolase.

Previous studies have demonstrated that enolases of various gram-positive and gram-negative bacteria bind plasminogen (reviewed in [4]), and this prompted us to investigate whether the enolase of *B. burgdorferi* was capable of binding human plasminogen. Microtiter plates were coated with recombinant enolase, bovine serum albumin (negative control protein) or ErpC (a known *B. burgdorferi* plasminogen-binding protein [10]), and then plasminogen binding was assayed by ELISA. Enolase demonstrated significant binding of human plasminogen (Figure 4A). Analyses using various concentrations of recombinant enolase demonstrated that *B. burgdorferi* enolase bound plasminogen in a dose-dependent manner, with an apparent K*_d_* of 125 nM (Figure 4B).

### Role of lysines in enolase-plasminogen binding

**Figure 5:**
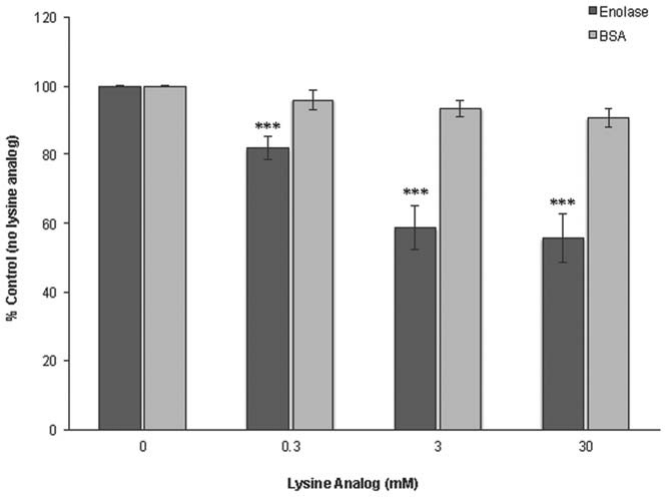
Role of lysines in enolase/plasminogen binding activity. Binding of plasminogen to immobilized enolase (10 µg/ml) was analyzed by ELISA. Plasminogen (25 µg/ml) was added to enolase-coated wells in the presence or absence of 0–30 mM ε-aminocaproic acid (lysine analog). Bound plasminogen was detected using a specific antiserum. BSA was used as a negative control. Data represent the means and standard errors from three separate experiments with 12 replicates per condition. ***, *P*<0.001, Student's *t* test assuming unequal variances.

Plasminogen receptors, both mammalian and bacterial, often bind plasminogen through lysine residues that interact with the Kringle domains of plasminogen [14], [39], [40], [41]. The enolase of *B. burgdorferi* is comprised of 5.7% lysine residues. Addition of the lysine analog ε-aminocaproic acid significantly reduced the interaction between enolase and plasminogen, while the lysine analog had no effect on the background binding of plasminogen to BSA (Figure 5). These data indicate a role for lysines in enolase-plasminogen binding.

### Role of ionic interactions in enolase-plasminogen binding

**Figure 6:**
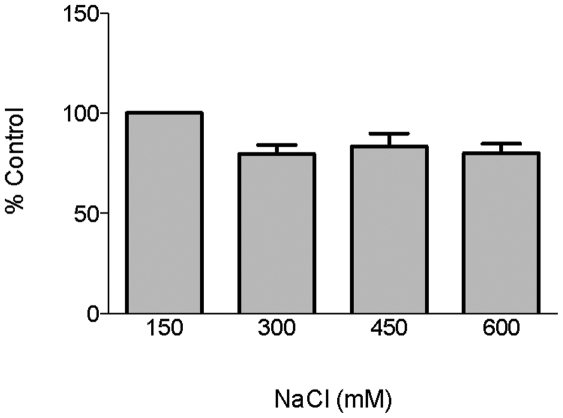
Role of ionic interactions in enolase binding of plasminogen. Binding of plasminogen to immobilized enolase (10 µg/ml) was analyzed by ELISA. Plasminogen (25 µg/ml) was incubated in the presence of increasing concentrations of NaCl. Bound plasminogen was detected using a specific antiserum. BSA was used as a negative control. Data are presented as % control (physiological NaCl, 150 mM) from the means and standard errors from three experiments with six replicates per concentration of NaCl.

The enolase of *B. burgdorferi* is highly charged, with a theoretical pI of 5.38. To assess the role of ionic interactions in enolase binding of plasminogen, binding assays were performed in the presence of various concentrations of NaCl. NaCl at up to four times the normal physiological concentration had a small but insignificant negative effect on binding (Figure 6). These data suggest that ionic interactions are not required for the enolase-plasminogen interaction.

### Plasminogen bound to enolase can be converted to active plasmin

**Figure 7:**
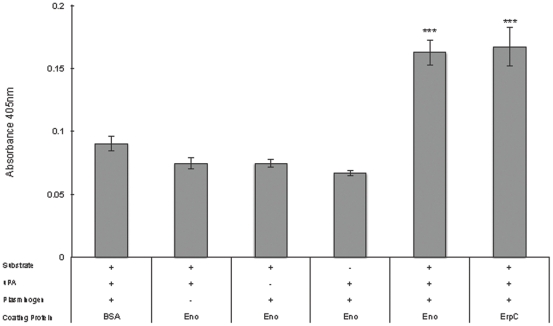
Enolase-bound plasminogen is converted into plasmin. Enolase-coated wells of microtiter plates were incubated with plasminogen, urokinase (uPA), and/or a plasmin-specific chromogenic substrate. Proteolytic activity was measured by absorbance at 405 nM. Data represent the means and standard errors from three different experiments with six replicates per condition. ***, *P*<0.001 compared to the activation of plasminogen bound to the control protein, BSA, Student's *t* test assuming unequal variances.

*B. burgdorferi*-bound plasminogen is converted to plasmin [11]. To determine if enolase-bound plasminogen can also be converted to the active enzyme, microtiter plates were coated with enolase, blocked, plasminogen and the activator uPA were added, and proteolytic activity was quantified using a plasmin-specific chromogenic substrate. As shown in Figure 7, enolase-bound plasminogen was converted to active plasmin. These data suggest that, *in vivo*, enolase-bound plasminogen would be accessible to plasminogen activators.

## Discussion

Enolases belong to a class of proteins referred to as moonlighting proteins: proteins that have multiple functions and often exist in distinct locations within a cell [42]. Along with its role as an essential glycolytic enzyme, we have demonstrated that *B. burgdorferi* enolase has an additional function as a cell-surface plasminogen receptor. Enolases lack traditional sorting signals and how these proteins become surface exposed is unknown. The enolase of *B. burgdorferi* is both cytoplasmic and surface exposed (Figures 2 and 3, and [36]), and humans and animals produce antibodies to the *B. burgdorferi* enolase during natural infection [43]. Proteins destined for the outer surface are expensive for a cell to produce [44], and the amino acid sequence of enolase certainly suggests an inexpensive protein. The *B. burgdorferi* protein, like many enolases, is rich in “cheap” amino acids such as alanine (8.3%) and glycine (7.2%) while low in ‘expensive’ amino acids such as phenylalanine (3.9%) and tryptophan (1.3%). Intriguingly, a hydrophobic domain in human αenolase suggested to play a role in its membrane association (AAVPSGASTGI) is identical in the *B. burgdorferi* enolase [14]. It is clear, however, that for both eukaryotic and bacterial enolases, surface exposure is not unusual [16], [27], [28], [29], [30], [32], [33], [34], [35], [45]. Other cytoplasmic proteins, such as DnaK, have also been identified as plasminogen-binding proteins [46], [47].

A potential consequence of the surface exposure of the *B. burgdorferi* enolase is its possible contribution to autoimmunity. Streptococcal enolases are cross-reactive with human enolase and are thought to play a role in post-streptococcal sequelae such as rheumatic heart disease [48]. Eukaryotic enolases are also associated with autoimmune conditions. The identified cross-reactive epitopes in cancer-associated retinopathy (FRAAVPSG and RYMGKGVS) are almost identical to the residues present in the *B. burgdorferi* enolase [49].

Lysine residues play a critical role in the interaction of plasminogen receptors with their ligand. A number of bacterial plasminogen-binding proteins contain essential lysines [4], [6], [10], [50]. The lysine analog ε-aminocaproic acid significantly inhibited enolase binding to plasminogen. For many plasminogen receptors, it is the C-terminal lysines that are critical for binding [16], [39]. The lysine residues in *B. burgdorferi* enolase, however, are dispersed. Internal plasminogen binding sites have been identified for other pathogen's receptors [51], [52], [53]. We are continuing to define the plasminogen-binding site(s) of the *B. burgdorferi* enolase.

Plasminogen binding by *B. burgdorferi* is clearly important *in vivo.*

Plasminogen-deficient mice exhibit reduced spirochetemia, and plasminogen is required for efficient dissemination of *B. burgdorferi* within the tick vector [9]. *B. burgdorferi*-bound plasminogen can degrade extracellular matrix proteins and activate matrix metalloproteases, facilitating the spirochete's spread through host tissues [6], [7], [8]. Interestingly, *B. burgdorferi* upregulates expression of both the plasminogen activator uPA and the plasmin inhibitor PAI-2 from monocytes. The upregulation of uPA could activate plasminogen bound to the surface of *B. burgdorferi*. The upregulation of PAI-2 had no effect on *B. burgdorferi* transmigration but did decrease monocyte migration across Matrigel [54]. In addition to the suppression of leukocyte function, *B. burgdorferi* may capitalize on plasminogen binding for other immune evasive strategies. For instance, binding of plasminogen is anti-opsonic for *Staphylococcus aureus*, and the enolase of *Streptococcus sobrinus* increases IL-10 expression in mice [55], [56].

It is not surprising that *B. burgdorferi* may express multiple plasminogen-binding proteins on its surface [10], [11], [12], [13]. Redundancy in bacterial adhesins is not unusual. Indeed, *B. burgdorferi* possesses at least three distinct fibronectin-binding proteins [57], [58], [59]. Many bacterial pathogens possess both high and low-affinity plasminogen-binding proteins on their cell surface [60], [61]. The *B. burgdorferi* plasminogen-binding protein ErpP, for instance, has a Kd of ∼25 nM, while we calculate that the enolase has a Kd of ∼125 nM (Figure 4B, and [10]. In addition, *B. burgdorferi* may utilize different plasminogen-binding receptors at different stages in its infectious life cycle, i.e., tick versus mammal. Plasminogen binding may not be the only function of extracellular enolase; the enolase of *S. aureus* binds laminin, and LenA, a surface receptor of *Leptospira interrogans*, interacts with plasminogen, complement Factor H, and laminin [26], [31], [62]. The plasminogen-binding OspA protein of *B. burgdorferi* also functions in invasion of the tick salivary glands, and ErpP of *B. burgdorferi* binds both plasminogen and complement factor H [10], [63].

*B. burgdorferi* is a successful extracellular pathogen that co-opts host proteins for its own advantage. We have identified that the enolase of *B. burgdorferi* moonlights as a surface-exposed, plasminogen-binding protein.
